# Effect of reduced phosphorus fertilizer application on the physical properties and chemical composition of 15 roasted tobacco leaves

**DOI:** 10.1515/biol-2025-1249

**Published:** 2026-03-12

**Authors:** Shihang Huang, Li Zhang, Jiancai Qian, Yongliang Han, Feng Tian, Xixian Ou, Xinglong Fan, Qiang Li

**Affiliations:** Jiangsu China Tobacco Industry Limited Liability Company, Nanjing, China; Hunan Agricultural University, Changsha, Hunan Province, China

**Keywords:** tobacco, phosphate fertilizer, physical characteristics of tobacco, chemical composition of tobacco

## Abstract

Phosphorus, as a crucial element affecting the physical properties and chemical quality of tobacco leaves, is often used excessively in tobacco production, leading to a prolonged surplus in the soil that poses risks to both the environment and tobacco quality. Despite its importance, there is limited research on the variations in phosphorus levels among tobacco leaves positioned differently in high-phosphorus soils. Therefore, investigating the rational reduction of phosphorus fertilizers in phosphorus-rich soils is essential. This study was conducted in the Guiyang Tobacco Growing Area of Chenzhou, Hunan Province, China. The widely planted Yunyan 87 tobacco variety was utilized and three phosphorus levels were created, including CK representing farmers’ habitual phosphorus fertilizer treatment (P_2_O_5_ 139.65 kg ha^−1^), P1 reducing phosphorus by 25 % with base fertilizer (P_2_O_5_ 104.85 kg ha^−1^), and P2 reducing phosphorus by 50 % with base fertilizer (P_2_O_5_ 69.9 kg ha^−1^). The effects of phosphorus dosage on the physical properties and chemical quality of tobacco were investigated. In the phosphorus-rich soils, the conventional phosphorus application (CK) was optimal for the 5th, 6th, 7th, and 10th leaves; reducing phosphorus by 25 % with basal fertilizer (P1) was optimal for leaves 7–12; and reducing phosphorus by 50 % with base fertilizer (P2) was optimal for leaves 9–11. The excessive phosphorus application could decrease the coordination between the physical properties and chemical composition of tobacco leaves, particularly affecting the leaf thickness, single-leaf weight, total sugar, and reducing sugar. However, reducing the base and phosphorus fertilizer application by 25 % improved multiple indicators of tobacco leaf quality, aligning better with high-quality tobacco standards in the region.

## 1 Introduction

Flue-cured tobacco, as a significant economic crop cultivated in countries such as China, the United States, and Brazil, prioritizes both the economic yield and quality of tobacco leaves [1]. The quality of these leaves is closely related to cigarette quality, as their characteristics directly affect the final cigarette product [2]. The chemical composition of tobacco is a fundamental determinant of leaf quality and characteristics [3], with the physical properties and chemical composition serving as the critical indicators in tobacco quality evaluation. Various factors such as the ecological environment, climate conditions, tobacco varieties, and soil fertility affect both yield and quality [4]. Precision fertilization is a key method for enhancing tobacco leaf quality during agricultural production [5].

Phosphorus (P) is an essential nutrient for plant growth, functioning, and reproduction [6], comprising 0.15 %–0.6 % of the dry weight of tobacco. It significantly influences the growth, development, and genetic metabolism of tobacco, as well as the quality of tobacco leaves [7], 8]. However, phosphorus limitation in agricultural ecosystems is a global issue, with most of the phosphorus in soil, such as organic, adsorbed, and mineral-bound phosphorus, not easily utilized by plants [6], 9]. This problem is exacerbated in southern China, where severe soil leaching, desilication, iron enrichment, and aluminum mineralization contribute to phosphorus fixation [10]. To ensure yield, farmers apply large amounts of phosphorus, which increases the soil phosphorus accumulation and leads to a widespread surplus in agricultural systems, contributing to a global peak in phosphorus development and decreased soil phosphorus utilization efficiency (PUE) [11]. The excessive phosphorus accumulation can result in soil compaction and increased salt content, which affects sustainable agriculture, crop yield, and quality [12], 13]. Therefore, it is essential to optimize the phosphorus fertilizer application rates to minimize the environmental damage and adverse effects.

The south of China is a major tobacco-producing region, with Guiyang County in Chenzhou City, Hunan Province being the largest tobacco-producing county in the province and a renowned “tobacco warehouse” nationally. This county leads the province in the tobacco area and ranks second nationally in output. Currently, the high phosphorus content in local soil has resulted in the surplus phosphorus and decreased utilization efficiency owing to farmers’ long-standing reliance on experience-based fertilization practices with relatively high phosphorus application. Consequently, there is an urgent need to determine the appropriate phosphorus levels for scientific fertilization. This study aimed to (1) examine the changes in the physical characteristics of tobacco leaves at positions 1–15 following the reduced phosphorus fertilizer application and (2) analyze the changes in the chemical composition at the same leaf positions and their impact on the coordination of the chemical composition. These findings could help to understand the effects of reduced phosphorus fertilizer application on tobacco quality and offer scientific advice for local phosphorus fertilizer management.

## 2 Materials and methods

### 2.1 Description of the study area

The study was conducted in 2023 at a representative tobacco cultivation site in Guiyang County (112°13′26“-112°55′46” E, 25°27′15“-26°13′30” N), Chenzhou City, southeastern Hunan Province, China. The region features the yellow soil and a humid subtropical monsoon climate with four distinct seasons: cold and dry winters and hot, rainy summers. The average annual temperature ranges from 15 °C to 23 °C, with 1,527.8 h of sunshine and 1,485.5 mm of precipitation. The pre-experiment soil physicochemical properties were as follows: pH 7.09, organic matter 47.71 g/kg, total nitrogen 2.05 g/kg, alkaline dissolved nitrogen 142.10 mg/kg, total phosphorus 1.75 g/kg, quick-acting phosphorus 87.63 mg/kg, and quick-acting potassium 412.15 mg/kg. The land use type was the tobacco-rice rotation.

### 2.2 Experimental design and management

The experiment was performed using three phosphorus levels: CK representing the tobacco farmers’ habitual phosphorus fertilizer treatment (P_2_O_5_ 139.65 kg ha^−1^); P1 with 25 % phosphorus reduction in basal fertilizer (P_2_O_5_ 104.85 kg ha^−1^); and P2 with 50 % phosphorus reduction in basal fertilizer (P_2_O_5_ 69.9 kg ha^−1^). All treatments included 154.5 kg ha^−1^ of nitrogen fertilizer and 440.4 kg ha^−1^ of potassium fertilizer. The field plot experiments were conducted on plots measuring 8 m × 6.47 m (51.76 m^2^), with three repetitions for each treatment arranged randomly in groups.

The tobacco variety used in this study was Yunyan 87, and the detailed fertilization plan is shown in Table 1.

**Table 1:** Fertilization plan.

	Base fertilizer (February 20, 2023)	Seedling fertilization (March 23 and April 3, 2023)	Topdressing (April 11th, April 23rd, and May 5th, 2023)
CK	Tobacco specific base fertilizer: 750 kg ha^−1^	Tobacco specific seedling fertilizer: 135 kg ha^−1^	Tobacco specific topdressing: 750 kg ha^−1^ K_2_SO_4_ : 300 kg ha^−1^
P1	Tobacco specific base fertilizer: 562.95 kg ha^−1^ 5Ca(NO_3_)_2_·NH_4_NO_3_·10H_2_O: 87.45 kg ha^−1^ K_2_SO_4_ : 30 kg ha^−1^	Tobacco specific seedling fertilizer: 135 kg ha^−1^	Tobacco specific topdressing: 750 kg ha^−1^ K_2_SO_4_ : 300 kg ha^−1^
P2	Tobacco specific base fertilizer: 375 kg ha^−1^ 5Ca(NO_3_)_2_·NH_4_NO_3_·10H_2_O: 175.05 kg ha^−1^ K_2_SO_4_ : 60 kg ha^−1^	Tobacco specific seedling fertilizer: 135 kg ha^−1^	Tobacco specific topdressing: 750 kg ha^−1^ K_2_SO_4_ : 300 kg ha^−1^

Tobacco specific base fertilizer (containing 7 % N, 17 % P_2_O_5_, 8 % K_2_O, and 15 % organic matter); Tobacco specific seedling fertilizer (containing 20 % *N* and 9 % P_2_O_5_); Tobacco specific topdressing (containing 10 % *N* and 32 % K_2_O); K_2_SO_4_ (K_2_O = 52 %, S = 17 %); and 5Ca(NO_3_)^2^·NH_4_NO_3_·10H_2_O (*N* = 15 %). All tobacco specific fertilizers used in this experiment is produced by Hunan Jinye Zhongwang Technology Co., Ltd.

### 2.3 Sample collection and measurement indicators

During the dome stage of tobacco production, 10 representative plants with consistent growth and no pests or diseases were selected from each plot (after topping and removing the base leaves). Different colored yarn markers were placed on each of the 15 leaves from top to bottom for subsequent harvesting and baking. Following tobacco harvesting and baking, physical characteristic indicators, including leaf length, leaf width, single leaf weight, equilibrium moisture content, thickness, and leaf mass weight, were measured. After assessing the physical properties, the tobacco samples were ground into fine powders for the analysis of chemical composition, including total sugar, reducing sugar, total nitrogen, nicotine, potassium, water-soluble chlorine, starch, and protein.

### 2.4 Measurement method

The measurement of physical properties was conducted in the following order, with all test indicators selected from the same batch of leaves.

(1) After balancing the moisture content of tobacco leaves for 7 days, the moisture content of tobacco is about 12 %. Weigh the total weight of 10 tobacco leaves using a 1/1,000 analytical balance and divide the result by 10 to obtain the weight of each leaf.
(2) The length and width of each leaf was measured using a soft ruler and calculated the average length and width from 10 leaves.
(3) A circular punch with a diameter of 1.5 cm was used to process the selected 10 tobacco leaves, extracting one circular disc from each leaf while avoiding leaf veins as much as possible. The total thickness of the 20 small discs was measured using a thickness gauge, and the average thickness of these discs was employed as the sample thickness.
(4) A total of 20 small discs was weighed, leaf mass weight (g/m^2^) = average disc weight ÷ disc area × 10,000.
(5) A total of 20 small discs was placed in an oven and dried for 6–8 h. Once the sample reached equilibrium weight, it was weighed again. Balanced moisture content (%) = (weight of 20 discs–dry weight after drying) ÷ weight of 20 discs × 100 %.

The chemical composition was determined as follows. Total sugar, reducing sugar, total nitrogen, nicotine, water-soluble chlorine, starch, and protein were measured using an AA3 continuous flow analyzer, while potassium was determined using flame spectrophotometry [14].

### 2.5 Data analysis

All the data were organized using Microsoft Excel. The significance (P < 0.05) of differences in the mean values (3 replicates) of various indicators between different treatments was examined using one-way ANOVA and the Least Significant Difference (LSD) test in SPSS 25.0 (IBM, USA). All images were created using the Origin 2022 software.

## 3 Results

### 3.1 Effect of phosphorus fertilizer reduction on the physical characteristics of roasted tobacco

**Figure 1:**
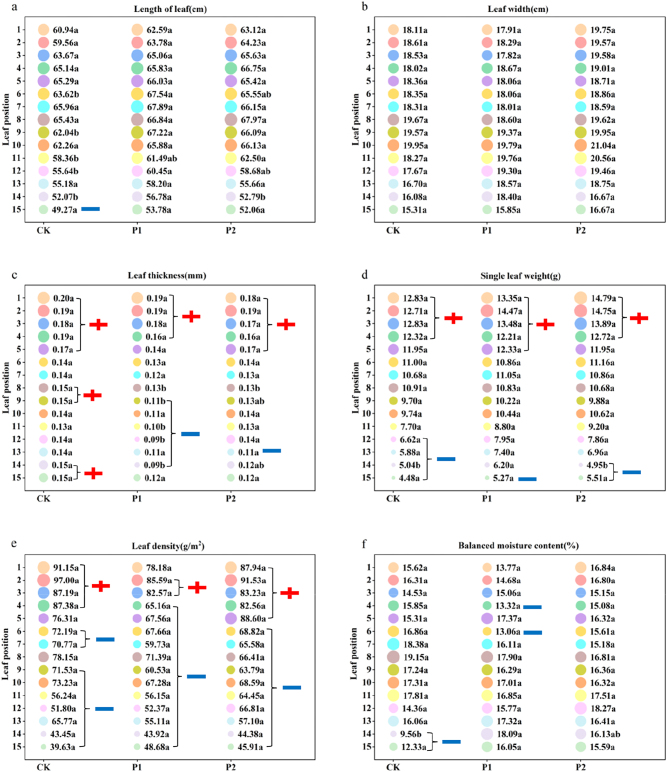
Effects of different phosphorus fertilizer rates on physical characteristics (leaf length (a), leaf widthc (b), leaf thickness (c), single leaf weight (d), leaf mass weight (e), and balanced water content (f)) of roasted tobacco. Note: CK, tobacco farmer’s habitual phosphorus fertilizer treatment (P_2_O_5_ 139.65 kg ha^−1^); P1, basal fertilizer with reduced phosphorus by 25 % (P_2_O_5_ 104.85 kg ha^−1^); P2, basal fertilizer with reduced phosphorus by 50 % (P_2_O_5_ 69.9 kg ha^−1^); 1–15 are corresponding to each leaf above and below the cigarette plant. Data is presented as mean of three replicates (*n* = 3). Statistical significance (p < 0.05) between treatments was determined by one-way ANOVA with LSD post hoc test. Plus sign means that this value is beyond the appropriate range, minus sign means that this value is below the appropriate range, and unlabeled are values within the appropriate range.

As shown in Figure 1, significant differences were observed in the physical characteristics at different phosphorus application rates.

From the perspective of “leaf length”, the trends for the 6th and 12th leaves were the same, with P1 > P2 > CK. The length of the 9th leaf in the CK treatment was significantly lower than that in the other treatments, whereas the length of the 11th leaf significantly increased with reduced phosphorus fertilizer. The 14th leaf in the P1 treatment was significantly longer than that in the other treatments. Under the CK, the 15th leaf was below the suitable range, whereas the remaining leaf positions were within the suitable range. In contrast, the 15th leaves of both treatments with reduced phosphorus were within the appropriate range, with all leaf positions falling within the suitable range.

There was no significant difference in “leaf width” among the three treatments across all leaf positions.

In terms of “leaf thickness”, the thickness of the 8th leaf in the CK treatment was significantly higher than that in the other treatments. The 9th and 14th leaves followed the trend CK > P2 > P1, whereas the 11th and 12th leaves demonstrated P1 as significantly lower than the others. In the CK treatment, the 8th, 9th, 14th, and 15th leaves were all beyond the suitable range, whereas the 6th, 7th, and 10th–13th leaves were within the suitable range. For P1, the leaves 1–4 were beyond the suitable range, the leaves 9–14 were below it, and the leaves 5–8 and 15 were within the suitable range. In P2, the leaves 1–5 were beyond the suitable range, the 13th leaf was below it, and the 6th, middle leaves (7−12), and leaves 14 and 15 were within the suitable range. Reducing the phosphorus fertilizer resulted in no significant changes in the thickness of the upper leaves, except for the 5th leaf in P1, which became suitable. With a 50 % reduction in phosphorus, all middle leaves were within the suitable range, and the 14th and 15th leaves shifted from slightly thicker in the CK to suitable. This indicated that reducing the phosphorus fertilizer by 50 % significantly affected the thickness of tobacco leaves.

Regarding “single-leaf weight”, the 14th leaf in P1 was significantly heavier than that in the other treatments, with no significant differences in other leaf positions. In the CK treatment, the leaves 1–4 were above the suitable range, the leaves 12–15 were below it, and the leaves 5–11 were within the suitable range. For P1, the leaves 1–5 were above the suitable range, the leaf 15 was below it, and the leaves 6–14 were within the suitable range. In P2, the leaves 1–4 were above the suitable range, the leaves 14 and 15 were below it, while the leaves 5–13 were within the suitable range. Reducing the phosphorus fertilizer by 25 % or 50 % caused the 12th and 13th leaves to transition to the appropriate range. This indicated that reducing the phosphorus fertilizer increased the number of middle and lower leaves with a single leaf weight within the appropriate range. Additionally, reducing the phosphorus fertilizer by 25 % significantly increased the single leaf weight of the 14th leaf to the appropriate range, but caused the 5th leaf to exceed it.

There was no significant difference in “leaf mass weight” among all leaf positions in the three treatments. In the CK treatment, leaves 1–4 exceeded the suitable range, leaves 6, 7, and 9–15 were below it, and leaves 5 and 8 were within the suitable range. In P1, leaves 2 and 3 were beyond the suitable range, leaves 4–15 were below it, and the first leaf was within the suitable range. For P2, leaves 1–5 were beyond the suitable range, leaves 6–15 were below it, and no leaf positions were within the suitable range. The reduction of phosphorus fertilizer led to a gradient decrease in leaf mass weight for the 8th leaf, falling below the suitable range. The phosphorus reduction also caused the 5th leaf weight to fall below or above the suitable range. Reducing the phosphorus fertilizer by 25 % brought the weight of the first leaf to the appropriate range, but further reduction (by 50 %) caused it to exceed the appropriate range again. This indicated that reducing the phosphorus fertilizer did not improve the weight of tobacco leaves.

Regarding “balance moisture content”, there was no clear pattern between CK and P1, and no significant change in the P2 treatment from top to bottom. For the 14th leaf, the order was P1 > P2 > CK, with no significant differences in other leaf positions. In CK, the leaves 14 and 15 were below the suitable range, while the leaves 1–13 were within it. In P1, the leaves 4 and 6 were below the suitable range, while the leaves 1–3, 5, and 7–15 were within the suitable range. All leaf positions in P2 were within the appropriate range. Reducing the phosphorus fertilizer increased the equilibrium moisture content of the 14th and 15th leaves to the appropriate range, whereas the 25 % reduction lowered the moisture content of the leaves 4 and 6 below the appropriate range. This indicated that a 50 % reduction in phosphorus fertilizer had a beneficial effect on the moisture content of tobacco leaves.

Considering all the physical indicators, reducing the phosphorus fertilizer application resulted in increased leaf length as the phosphorus levels decreased. The leaf thickness in P2 was generally suitable, and the single leaf weight in P1 and P2 increased by two leaves within the appropriate range compared with CK. P2 provided the optimal equilibrium moisture content. There were no significant changes in the leaf width or mass weight. Overall, the physical properties of tobacco improved after phosphorus reduction.

### 3.2 Effect of phosphorus fertilizer reduction on the chemical composition of roasted tobacco

#### 3.2.1 Chemical composition

**Figure 2:**
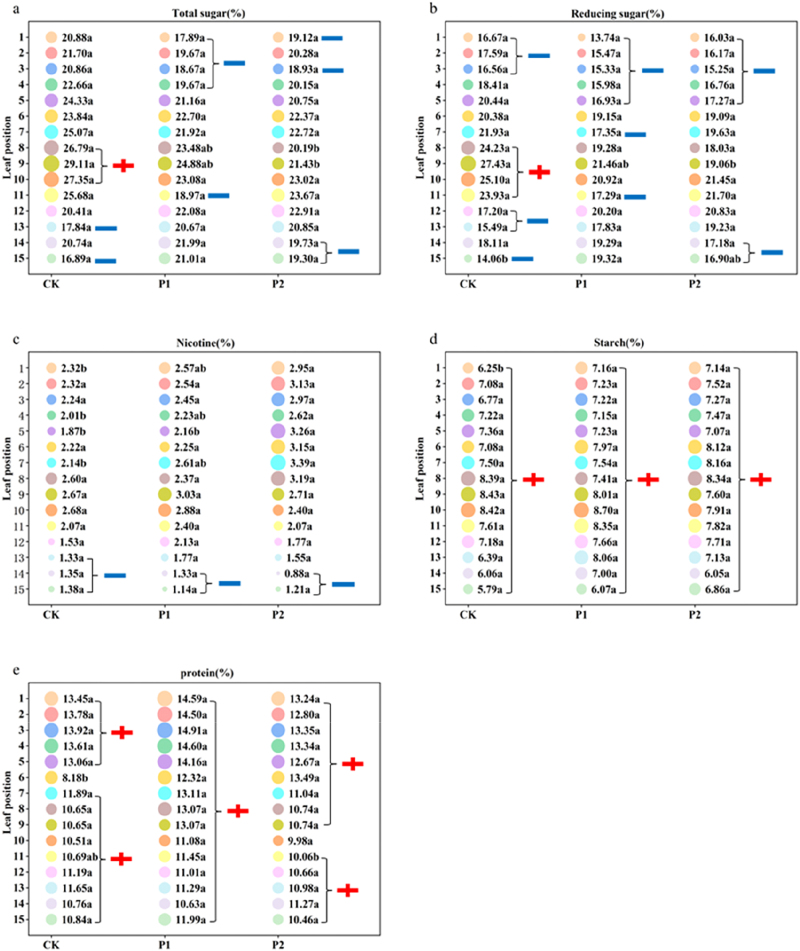
Effects of different phosphorus fertilizer dosages on the chemical composition (1) of roasted tobacco (total sugars (a), reducing sugars (b), nicotine (c), starch (d), and protein (e)). Note: Same as Figure 1.

**Figure 3:**
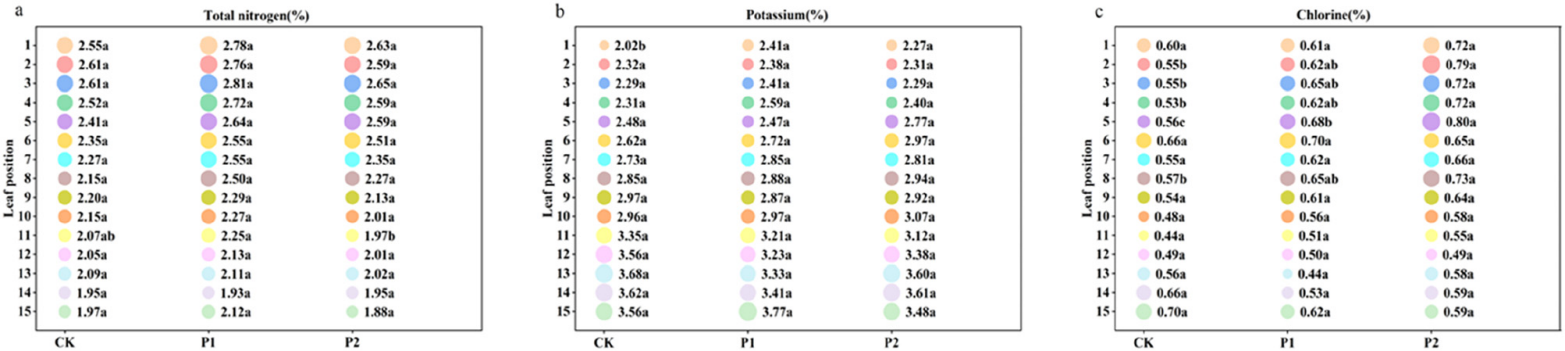
Effects of different phosphorus fertilizer dosages on the chemical composition (2) of roasted tobacco (total nitrogen (a), potassium (b), and chlorine (c)). Note: Same as Figure 1.

Figures 2 and 3 illustrate the significant differences in the chemical composition among the treatments with varying phosphorus application rates.

Regarding “total sugar”, the 8th and 9th leaves significantly decreased with the reduced phosphorus application, following the order CK > P1 > P2, whereas no significant differences were observed in other leaf positions. In CK, the 13th and 15th leaves were below the suitable range; the 1st to 7th leaves and the 11th, 12th, and 14th leaves were within the suitable range; and the 8th to 10th leaves were above the suitable range. For P1, the 1st to 4th and 11th leaves were below the suitable range, whereas the 5th to 10th and 12th to 15th leaves were within the suitable range. In P2, leaves 1, 3, 14, and 15 were below the suitable range, whereas leaves 2 and 4–13 were within it. Reducing phosphorus fertilizer caused the first and third leaves to decrease from the range to below it (P1 and P2), and the 8th and 10th leaves to drop from beyond the range to below it (P1 and P2). Reducing the phosphorus by 25 % lowered the 2nd, 4th, and 11th leaves below the suitable range, while increasing the 13th and 15th leaves to the suitable range. A further reduction of 50 % caused the 14th leaf to fall below the appropriate range.

For “reducing sugar”, the 9th leaf exhibited a significant decrease with reduced phosphorus dosage, following the order CK > P1 > P2, and the 15th leaf presented P1 > P2 > CK. In CK, the 1st to 3rd leaves and the 12th, 13th, and 15th leaves were below the suitable range; the 4th to 7th leaves and the 14th leaf were within the suitable range; and the 8th to 11th leaves were above the suitable range. For P1, the 1st to 5th leaves and the 7th and 11th leaves were below the suitable range, whereas the 6th, 8th to 10th, and 12th to 15th leaves were within the suitable range. In P2, the 1st to 5th leaves and the 14th and 15th leaves were below the suitable range, whereas the 6th to 13th leaves were within this range. Reducing the phosphorus fertilizer caused the 4th and 5th leaves to decrease from the range to below it (P1 and P2), the 8th and 10th leaves to drop from beyond the range to below it (P1 and P2), and the 12th and 13th leaves to increase from below the range to below it (P1 and P2). With a 25 % reduction in phosphorus, the 7th leaf fell below the suitable range, the 15th leaf rose to the suitable range, and the 11th leaf decreased from above the range to below it. A 50 % reduction in phosphorus brought the 11th leaf within the suitable range but caused the 14th leaf to fall below it.

From the perspective of “nicotine”, the trend in leaves 1, 4, and 7 was consistent, with P2 > P1 > CK. Notably, P2 in the fifth leaf exhibited a significantly higher nicotine content than the other treatments. Leaves 13–15 in CK and leaves 14 and 15 in P1 and P2 fell below the range. However, after reducing the phosphorus fertilizer, the nicotine content in the 13th leaf of both phosphorus reduction treatments increased to within the range.

From the perspective of “Starch”, the starch content in the first leaf of CK was significantly lower than that in the other treatments. The starch content in this region of tobacco exceeded the suitable range, and no significant change in tobacco starch content was observed with the reduced phosphorus fertilizer.

From the perspective of “protein”, in the 6th leaf, CK exhibited significantly lower protein levels than the other treatments, whereas in the 11th leaf, the protein content followed the order: P1 > CK > P2. The 6th leaf of CK and the 10th leaf of P2 were within it, whereas no leaf position of P1 fell within this range. After reducing the phosphorus fertilizer, the protein content in the 6th leaf of both phosphorus reduction treatments increased beyond it, whereas reducing phosphorus by 50 % brought the 10th leaf into suitable range.

From the perspective of “Total nitrogen”, in the 11th leaf, the order of total nitrogen content was P1 > CK > P2, with no significant differences observed in other leaf positions. All the leaf positions across the three treatments fell within the suitable range. Reducing the application of phosphate fertilizer had almost no effect on the nitrogen content of tobacco leaves.

For “Potassium”, the content in the first leaf of CK was significantly lower than that in the other treatments, while no significant differences were observed in the other leaf positions. All the leaf positions across the three treatments fell within the suitable range. Reducing the application of phosphate fertilizer had almost no effect on the potassium content of tobacco leaves.

From the perspective of “Chlorine”, the trends for the 2nd, 3rd, 4th, 5th, and 8th leaf positions were similar, with CK < P1 < P2. All the leaf positions in the three treatments were within the suitable range. Reducing the application of phosphorus fertilizer increased the chlorine content in many leaf positions.

Considering all the chemical components, reducing the application of phosphorus fertilizer led to a gradual decrease in total sugar and reducing sugar in some tobacco leaf positions. The nicotine content demonstrated no significant change overall, whereas it increased in some leaf positions with the reduced phosphorus fertilizer. The starch, protein, total nitrogen, and potassium levels remained unchanged, whereas the chloride ion levels increased in some leaf positions with the decreased phosphorus fertilizer. Although there could be slight variations in the chemical composition across the three treatments, it was unclear which treatment and leaf position were optimal. Therefore, calculating the chemical composition ratio and assigning scores to the chemical components are necessary for further exploration.

### 3.3 Chemical composition ratio

**Figure 4:**
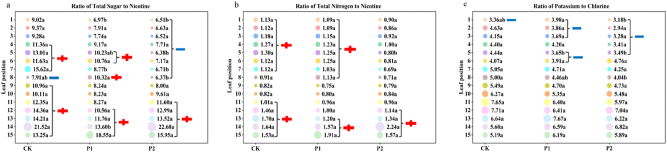
Effects of different phosphorus fertilizer dosages on the chemical composition ratios (ratio of total sugar (a), nitrogen-alkali ratio (b), and potassium-chlorine ratio (c)) of roasted tobacco. Note: Same as Figure 1.

Figure 4 shows significant differences in chemical composition ratios between treatments with different phosphorus application rates.

Regarding the sugar-alkali ratio, both CK and P1 exhibited a wavy trend from top to bottom, initially increasing, then decreasing, and increasing again, while P2 showed a gradual increase from the middle leaves. The 1st and 7th leaves of CK had significantly higher ratios than those of other treatments. For the 5th leaf, the ratio order was CK > P1 > P2, and for the 8th leaf, the order was P1 > CK > P2. The 14th leaf of P1 had a significantly lower ratio than that of the other treatments. In the CK, the leaves 4–7 and 9–15 exceeded the suitable range; the leaves 1–3 were within it; andthe leaves 8 were below it. In P1, the leaves 5, 6, 8, and 12–15 exceeded it, while the leaves 1–3 were below it. The leaves 4, 7, and 9–11 belonged to it. The leaves 1–8 of P2 are below the suitable range, the leaves 11–15 were above it, and only leaves 9 and 10 belonged to it. The phosphorus fertilizerwas reduced by 25 % or 50 %, with the sugar alkali ratio in the 9th and 10th leaves adjusted to the suitable range; the phosphorus was reduced by 25 % for the leaves 4, 7, and 11 to achieve the appropriate range. A further reduction of phosphorus by 50 % could cause the leaves 4 and 7 to fall below the suitable range, whereas the leaf 11 could rise above it.

From the perspective of the nitrogen alkali ratio, the P2 treatment of the fifth leaf was significantly lower than that of others, whereas no significant differences were observed in other leaf positions. In the CK treatment, the leaves 1–7 and 11–15 exceeded the suitable range, while the leaves 8–10 fall within it. For the P1 treatment, leaves 1–8 and 13–15 exceed the suitable range, while leaves 9–12 were within it. In the P2 treatment, leaves 12–15 exceed the suitable range, whereas leaves 1–11 were within it. After reducing phosphorus fertilizer, the nitrogen alkali ratio of the 11th leaf in both phosphorus reduction treatments fell to the appropriate range; with a 25 % reduction in phosphorus fertilizer, the 8th leaf’s ratio increased beyond the suitable range, while the 12th leaf’s ratio decreased to the suitable range; and with a 50 % reduction, leaves 1–7 were lowered to the appropriate range.

From the perspective of potassium chloride ratio, for leaf 1, the ratio was highest in P1, followed by CK and P2; the fifth leaf of CK showed a significantly higher ratio compared to other treatments; and for the 8th leaf, the ratio was highest in CK, followed by P1 and P2. The first leaf of CK was below the suitable range, while the remaining leaf positions were within it. In the P1 treatment, leaves 1–3, 5, and 6 were below the suitable range, whereas leaves 4 and 7–15 were within it. For the P2 treatment, leaves 1–5 were below the suitable range, while leaves 6–15 were within it. After reducing the phosphorus fertilizer, the potassium chloride ratio of the second, third, and fifth leaves in both phosphorus reduction treatments decreased to below the appropriate range; with a 25 % reduction in phosphorus fertilizer, the sixth leaf fell below the appropriate range; and with a 50 % reduction, the fourth leaf decreased to below the appropriate range.

Considering the ratios of the three chemical components, P1 exhibited the best sugar-to-alkali ratio, P2 had the best nitrogen-to-alkali ratio, and CK had the best potassium-to-chlorine ratio. Further comprehensive evaluation of these chemical indicators is required to assign values and scores.

### 3.4 Chemical composition assignment score

As shown in Table 2, the maximum score (100 points) for nicotine was ranked by leaf number as follows: P1 > CK > P2. For total nitrogen, the full score (100 points) was ranked by leaf number as CK > P1 > P2. The maximum score (100 points) for reducing sugars was ranked as P2 > P1 > CK. For potassium, the maximum score (100 points) was ranked as P1 = P2 > CK. The order of leaf numbers for starch above 50 min was CK > P1 = P2. CK achieved a full score (100 points) for the sugar alkali ratio (3), with one more leaf position than P1 (2), while P2 did not achieve a full score. The maximum score (100 points) for the nitrogen alkali ratio was ranked as P1 = P2 > CK. CK achieved a high potassium chloride ratio (2) with a score above 98 points, one leaf position more than P1 (1), and P2 did not achieve a high score.

**Table 2:** Effect of phosphorus fertilizer reduction on chemical composition index scores of roasted tobacco.

Treatment (sum of total points)	Leaf position	Nicotine	Total nitrogen	Reducing sugar	Potassium	Starch	Ratio of total sugar to nicotine	Ratio of total nitrogen to nicotine	Ratio of potassium chlorine	Total to weight score
CK (1,261.55)	1	100.00	95.00	93.35	90.40	56.25	100.00	94.64	50.40	89.73
	2	100.00	89.00	97.95	96.40	43.80	100.00	95.31	72.60	91.52
	3	100.00	89.00	92.80	95.80	48.45	100.00	91.34	62.80	89.76
	4	89.50	98.00	100.00	96.20	41.70	92.56	83.00	68.00	87.04
	5	83.50	100.00	100.00	99.60	39.75	79.90	80.00	68.80	82.91
	6	100.00	100.00	100.00	100.00	43.65	89.44	95.31	61.40	89.43
	7	97.00	100.00	100.00	100.00	37.50	81.50	95.31	80.50	88.22
	8	100.00	100.00	88.85	100.00	24.15	88.64	97.32	80.00	88.19
	9	100.00	100.00	65.70	100.00	23.55	93.76	91.34	84.90	85.97
	10	100.00	100.00	84.50	100.00	23.70	97.56	91.34	91.35	90.15
	11	93.50	100.00	90.35	100.00	35.85	86.50	100.00	98.25	89.52
	12	57.53	100.00	96.00	100.00	42.30	66.40	57.00	98.50	74.92
	13	50.47	100.00	87.45	100.00	54.15	67.90	42.00	93.20	71.60
	14	51.18	95.00	100.00	100.00	59.10	49.64	45.60	86.00	68.55
	15	52.23	97.00	80.30	100.00	67.10	77.50	52.20	81.90	74.04
P1 (1,284.87)	1	100.00	72.00	77.60	98.20	42.60	89.70	97.32	59.70	83.69
	2	100.00	74.00	87.35	97.60	41.55	96.10	97.32	57.90	86.55
	3	100.00	69.00	86.65	98.20	41.70	94.96	93.30	55.35	85.10
	4	100.00	78.00	89.90	100.00	42.60	100.00	87.00	64.00	87.92
	5	98.00	86.00	94.65	99.40	41.55	97.08	85.00	54.75	87.06
	6	100.00	95.00	100.00	100.00	30.45	92.96	85.00	58.65	87.55
	7	100.00	95.00	93.25	100.00	36.90	100.00	100.00	74.20	91.87
	8	100.00	100.00	100.00	100.00	38.85	96.72	94.64	69.20	91.54
	9	77.00	100.00	100.00	100.00	29.85	98.26	85.00	74.00	86.75
	10	92.00	100.00	100.00	100.00	19.50	98.92	90.00	83.50	90.15
	11	100.00	100.00	96.45	100.00	24.75	98.46	100.00	92.00	93.13
	12	96.50	100.00	100.00	100.00	35.10	95.76	100.00	92.05	93.09
	13	77.00	100.00	99.15	100.00	29.10	90.96	90.00	98.35	87.50
	14	50.47	93.00	100.00	100.00	45.00	46.44	49.80	92.95	67.55
	15	43.76	100.00	100.00	100.00	58.95	45.80	29.40	90.95	65.44
P2 (1,208.47)	1	85.00	87.00	90.15	95.40	42.90	85.10	96.65	47.70	81.74
	2	67.00	91.00	90.85	96.20	37.05	86.30	94.02	44.10	78.47
	3	83.00	85.00	86.25	95.80	40.95	85.20	100.00	49.20	81.09
	4	100.00	91.00	93.80	98.00	37.95	94.76	100.00	51.15	88.11
	5	54.00	91.00	96.35	100.00	43.95	83.80	90.00	52.35	77.50
	6	65.00	99.00	100.00	100.00	28.20	91.14	90.67	75.20	83.46
	7	41.00	100.00	100.00	100.00	27.60	87.10	79.33	65.00	76.25
	8	61.00	100.00	100.00	100.00	24.90	83.70	81.00	60.80	78.42
	9	100.00	100.00	100.00	100.00	36.00	96.65	89.00	74.40	91.17
	10	100.00	100.00	100.00	100.00	31.35	99.56	92.68	84.80	92.91
	11	93.50	97.00	100.00	100.00	32.70	91.60	100.00	89.70	90.89
	12	77.00	100.00	100.00	100.00	34.35	80.10	93.97	95.20	85.42
	13	58.24	100.00	100.00	100.00	43.05	74.80	74.00	91.10	78.95
	14	31.05	95.00	95.90	100.00	59.10	29.28	9.60	94.10	56.24
	15	46.23	88.00	94.50	100.00	47.10	56.24	49.80	88.90	67.85

Based on the total score for the weight of each leaf position in the chemical index scoring, the high-quality tobacco leaves with scores above 90 in the three treatments were as follows: CK: the 2nd and 10th leaves; P1: the 7th, 8th, and 10th-12th leaves. Among the 9th-11th leaves of P2, P1 had the most high-quality tobacco leaves. Although P2 had one more high-scoring leaf position (above 90) than CK, CK had a higher average score per leaf than P2. The second leaf in CK achieved the highest score at 91.52, the 11th leaf in P1 had the highest chemical index score at 93.13, and the 10th leaf in P2 had the best chemical index score at 92.91. The total weight scores were ranked as P1 > CK > P2, indicating that P1 exhibited the best chemical index coordination among all the leaf positions.

Overall, the high-quality tobacco leaves with excellent chemical composition coordination were more abundant in P1 than in the other treatments, and P1 also had the best combined score across all leaf positions.

## 4 Discussion

### 4.1 Physical characteristics

The physical properties of tobacco can help to evaluate the leaf quality, optimize the production processes, and are closely related to chemical composition. They may include certain factors such as leaf length, single-leaf weight, and thickness. The high-quality tobacco leaves should be at least 50 cm long to reflect their growth status [15]. The single leaf weight indicates both yield and quality. If it is too low, the leaves can be light and thin, whereas the too high weight can lead to reduced thickness and roughness, thereby lowering quality. In this study, the tobacco leaf length increased with the decreased phosphorus application, and the single-leaf weight of P1’s lower leaves significantly increased compared to that of CK. These changes may result from the excessive phosphorus application, which caused the greater crop respiration, reduced dry matter accumulation, and insufficient nutrient development, ultimately decreasing the yield. The ideal thickness for high-quality tobacco was approximately 130 μm; and the deviations from this could affect the quality [15]. The excessive phosphorus could cause the leaves to age prematurely and become thicker, resulting in the rough tissues that complicated processing and increased costs, while potentially affecting the quality and taste. Conversely, in Hunan Yongzhou K326 tobacco, the leaf thickness decreased with the increasing phosphorus application, while the leaf thickness of B2F and C3F in Hunan Yongzhou K326 and X2F remained unchanged. The phosphorus-rich soil in Enshi, Hubei, demonstrated no significant effects on the physical properties of Yunyan 87 across different phosphorus fertilizer application rates, with the minimal changes in leaf width, leaf mass weight, and moisture content.

### 4.2 Chemical composition

The chemical composition of tobacco leaves could provide an objective standard for assessing quality, as the main chemical components and their ratios directly affect tobacco quality. The total sugar and reducing sugar significantly affected the sensory quality of tobacco, with the high-quality leaves having a total sugar content between 20.00 % and 26.00 % and the reducing sugar content between 18.00 % and 22.00 %. The excessive sugar content could lower the pH of smoke, resulting in a dull and unpleasant taste and aroma, which complicated the processing and affected the quality. Conversely, the low sugar content could lead to the thermal decomposition of nitrogen compounds into alkaline products, stimulating the sensory expression. The phosphorus-rich soil in Enshi, Hubei Province, had little influence on nicotine, total sugar, and chlorine in Yunyan 87 across different phosphorus application rates. Increasing phosphorus application rates in Enshi led to higher reducing sugar levels in the upper leaves. In Ottawa, Canada, different phosphorus contents had no significant effect on the nicotine content of Hicks tobacco. This study showed that the trend in reducing sugars in the upper leaves was consistent with that observed in Hubei, and a similar trend was noted in the middle leaves. The total sugar content in the upper and middle leaves increased with the higher phosphorus fertilizer application, with P1 (25 % reduction in phosphorus) and P2 (50 % reduction in phosphorus) being more suitable for tobacco sugar content. Perhaps by continuing to apply phosphorus normally in high phosphorus soil, it maximizes photosynthetic efficiency, directs plants to prioritize the synthesis and accumulation of sugar, while reducing sugar consumption, resulting in an increase in sugar content in tobacco leaves. The different phosphorus application rates had no significant effect on nicotine, chlorine, starch, protein, total nitrogen, or potassium in Yunyan 87. Research has also indicated that reducing phosphorus fertilizer cannot necessarily affect the tobacco starch levels, depending on the variety or species [16]. The Ottawa experiment in Ontario, Canada, revealed that varying phosphorus content had no significant effect on nicotine content [17]. In this study, reducing the phosphorus fertilizer led to a slight increase in nicotine content in some upper and middle leaves, whereas all the treatments remained within the appropriate range and most leaf positions presented no significant changes. The chloride ion levels increased in some leaf positions with the reduced phosphorus fertilizer, while all the treatments remained within the appropriate range. Additionally, the starch content of the Yunyan 87 tobacco leaves did not exhibit the significant changes after the phosphorus reduction. Overall, the chemical component ratios of P1 and P2 were identified to be more suitable for the leaf positions than CK. The high-quality tobacco leaves with excellent chemical composition coordination were more prevalent in P1 than in the other treatments, and P1 also achieved the highest average chemical composition score across all leaf positions.

### 4.3 High quality tobacco leaves

In the large-scale tobacco production, the quality and economic benefits of the 4th–6th leaves and middle leaves (7th–12th leaves) were higher than those of other leaf positions. In this study, the leaves with a comprehensive chemical composition score of 90 or above included five leaves (7th, 8th, and 10th–12th) under the basal fertilizer with a 25 % phosphorus reduction (P1) treatment, three leaves (9th–11th) under the basal fertilizer with a 50 % phosphorus reduction (P2) treatment, and only one leaf (10th) under the CK treatment. Considering overall physical quality, the 5th, 6th, 7th, and 10th leaves of CK; the 7th–12th leaves of P1; and the 9th–11th leaves of P2 were classified as extremely high-quality tobacco leaves. This suggested that in the high-phosphorus soils, reducing the phosphorus fertilizer application was beneficial for achieving the higher quality tobacco leaves. In Tamil Nadu, India, 50 % of the conventional phosphorus application amount (22 kg/hm^2^) was recommended. Chewing tobacco could yield higher net income, improved leaf chemical properties, and greater phosphorus use efficiency [18]. In North Carolina, USA, reducing the phosphorus fertilizer did not significantly affect the tobacco plant height, yield, quality, or output value. However, it decreased the phosphorus fertilizer input, conserved the resources, reduced the fertilizer waste, lowered the carbon emissions from fertilizer production, and mitigated the risk of agricultural nonpoint source pollution [19]. These findings suggested that the effects of phosphorus reduction on tobacco leaves could vary depending on the variety, soil, and climate conditions. If reducing the phosphorus fertilizer did not significantly improve the tobacco yield, income, or quality, it may be advisable to temporarily reduce the phosphorus input. Overall, in the tobacco-growing area of Guiyang, Hunan, P1 (P_2_O_5_ 104.85 kg ha^−1^) was identified as a more suitable phosphorus application treatment.

## 5 Conclusions

In the Guiyang tobacco-growing area of Chenzhou, Hunan Province, CK was optimal for the 5th, 6th, 7th, and 10th leaves in this phosphorus-rich soil region. A 25 % reduction in phosphorus with basal fertilizer (P1) was optimal for the 7th–12th leaves, whereas a 50 % reduction (P2) was optimal for the 9th–11th leaves. The excessive phosphorus application led to the deterioration in the coordination between the physical properties and chemical composition of tobacco leaves. Reducing phosphorus by 25 % with basal fertilizer (P_2_O_5_ 104.85 kg ha^−1^) improved this coordination, meeting the standards for high-quality tobacco leaves in the region. Therefore, it can be advisable to appropriately reduce the phosphate fertilizer input to conserve the non-renewable phosphate resources and enhance the economic benefits.
